# Perceived Usability of Tablet Crushers: Comparison of Devices by People with and without Limited Hand Functions

**DOI:** 10.3390/pharmaceutics15020517

**Published:** 2023-02-03

**Authors:** Su Vin Lee, Tammy Aplin, Aida Sefidani Forough, Kathryn J. Steadman

**Affiliations:** 1 School of Pharmacy, The University of Queensland, Brisbane, QLD 4072, Australia; 2 School of Rehabilitation Sciences, The University of Queensland, Brisbane, QLD 4072, Australia; 3 Allied Health Research Collaborative, The Prince Charles Hospital, Brisbane, QLD 4032, Australia; 4 School of Clinical Sciences, Queensland University of Technology, Brisbane, QLD 4000, Australia

**Keywords:** dosage form modification, hand function, hand strength, pill crusher, swallowing difficulties

## Abstract

Tablet crushing is a common practice used by patients and their carers, mainly to facilitate swallowing. Various tablet-crushing devices with different designs are currently available on the market. This study aimed to compare the usability of different tablet-crushing devices in people with and without limited hand functions. The hand function of 100 adults recruited from the general community (40 of whom self-reported a limited hand function) was assessed using the hand and finger function subscale of the Arthritis Impact Measurement Scale version 2. The hand strength was measured using a dynamometer. Participants crushed tablets using 11 crushing devices and completed a Rapid Assessment of Product Usability and Universal Design questionnaire for each device. Hand-held twist-action crushers with an ergonomic grip received the highest usability scores among both groups, irrespective of the cost (*p* < 0.05). Crushers with bags were scored lower by those with limited hand functions, although the score improved if the device was automatic. Preferences regarding electronic crushers significantly changed once the cost was revealed. Economical twist-action crushers with ergonomic grips and without bags or cups were the most favoured crushers.

## 1. Introduction

Solid oral dosage forms such as tablets and capsules are the most common administration route for medications. Unfortunately, many people struggle to swallow solid oral dosage forms whole and instead cut or crush tablets or open capsules to make them easier to swallow [1]. People who have dysphagia, which is an impairment in the swallowing function leading to difficulties with safely swallowing food or liquids, find it necessary to modify their solid dose medications [2]. Members of the general public who are otherwise healthy and able to swallow food and liquids may also crush their tablets because they perceive them to be too large or difficult to swallow whole [3,4]. The proportion of the population who modifies their tablets in different settings varies, depending on the survey methods, from around 10–20% in the general population [3,4] to 18–80% in aged-care facilities and hospitals [5,6,7,8,9].

Tablet crushing can result in altered pharmaceutical properties, including a decreased therapeutic efficacy and an increased risk of drug instability. Certain types of tablets must not be crushed; for example, those with modified release characteristics because removing them can cause serious safety issues [6,7,10]. Additionally, when tablets are crushed, their film or sugar coatings are destroyed, which exposes the unpleasant taste of the ingredients. This results in non-compliance, which, in turn, causes therapeutic failure [1,5]. When managing patients who cannot swallow solid oral dosage forms, switching to an alternative liquid formulation or administration route is more suitable [1,11]. If there is no alternative available, crushing the solid oral doses may be considered [11,12]; most guidelines recommend the use of a pestle and mortar or a pill crusher [12].

There are a variety of tablet-crushing devices available from pharmacies or online, ranging from simple manual devices to large electronic devices. Most manual crushers function either by lifting a lever up and down to apply pressure to the crushing pad or by hand-twisting to create a rotating or grinding movement to crush the tablet within the crusher. A few devices use disposable cups or bags to reduce cross-contamination between users. Electronic crushers have an electronic button or a sensor-activated crush to minimise user fatigue. These devices are considered to be assistive products/devices, which means that they are purposed for facilitating medication modification for users [13]. However, these products are not anticipated to improve the health of users and, therefore, have no explicit legislation regulating them [13].

Despite various claims about the usefulness of these products, there is not much evidence regarding their actual usability in facilitating tablet crushing. The usability of a product refers to how it helps the user to perform a desired task. According to the ISO 9241–11 2018 standard [14], the main metrics of a usability assessment are effectiveness, efficiency, and user satisfaction in a specific context of use. The usability of crushing devices can be a big challenge, especially for people who have limited dexterity due to weakened muscle functions or the presence of a disability, e.g., older people or people with neuromuscular disorders, which may impact their independence in terms of medication management, as demonstrated for medication packaging [15].

The effectiveness of tablet-crushing devices can be measured in terms of the accuracy and completeness of the tablet crushing achieved by the users. Tablet crushers may vary in their performance in terms of the particle size produced [16]. Their effectiveness at minimising drug loss has also been shown to vary in the amount of drug loss; the reported drug loss from 24 different crushers ranged from 1.9 to 13.3% [17].

The efficiency of tablet-crushing devices can be discussed in the context of time efficiency, cost, and user fatigue. Nurses and other healthcare workers who frequently use such devices as a part of their routine medication practices may also be at risk of developing work-related musculoskeletal disorders (WMSD) such as musculoskeletal overexertion injuries if such devices are not comfortable to use [18].

Finally, user satisfaction or perceived usability are other important elements of a usability assessment. These can be reflected in the satisfaction with the features of tablet crushers as well as physical comfort and acceptability of use. However, user satisfaction remains to be understood for tablet crushers. In this study, we aimed to investigate the perceived usability of a wide range of commercially available tablet-crushing devices from people with and without limited hand functions.

## 2. Materials and Methods

This research complied with the tenets of the Declaration of Helsinki and was approved by the University of Queensland School of Pharmacy Ethics Committee (Reference Number 2016/13). Informed written consent was obtained from each participant prior to the study.

### 2.1. Study Participants

Participants aged over 16 years were eligible for inclusion. In the first phase of recruitment, participants without limited hand functions were enrolled. The participants were recruited from the staff, students, and visitors of the University of Queensland School of Pharmacy building and surrounding facilities through email, poster, and leaflet invitations. The second phase of the recruitment focussed on participants with limited hand functions and who were recruited through email invitations sent to Brisbane-based support groups for people with conditions likely to be associated with a limited hand function. Sessions were held during a monthly support group meeting for Arthritis Queensland and the Stroke Foundation, during which participants were recruited, consent was given, and data was collected. The demographic information was collected from each participant, including the age, gender, level of education, and current personal income. The participants were also asked whether they took any regular or occasional medications and whether they crushed pills before swallowing as well as any preferred method for crushing.

### 2.2. Assessment of Hand Function

The hand function was assessed in three ways. First, the participants were required to self-report any medical conditions that could impact the flexibility, strength, or movement of either or both of their hands. Second, the physical domain questions from the revised version of the Arthritis Impact Measurement Scales version 2 (AIMS2) were used to calculate a hand function score [19] due to the relevance of the questions to the use of the crushing devices. The questions asked about the ability to perform five tasks during the previous month on a 5-point Likert scale ranged from all days to no days. These tasks included writing with a pen or pencil, buttoning a shirt or blouse, turning a key in a lock, tying a knot or bow, and opening a new jar of food (Supplementary Materials). The responses to each task were allocated points from 1 for ‘all days’ to 5 for ‘no days’. The scores were then normalised to lie in a range of 0 to 10 [19]. A score of 0 represented good hand and finger functions whereas a score of 10 indicated poor hand and finger functions. As the third method of the hand function assessment, the hand strength was measured by squeezing a hydraulic hand dynamometer (Jamar, Brisbane, Australia) as tightly as possible [20]. The hand strength readings were made with participants seated in a uniform position; these were obtained twice for both hands and the average for each hand was used.

### 2.3. Assessment of Crusher Usability

**Figure 1:**
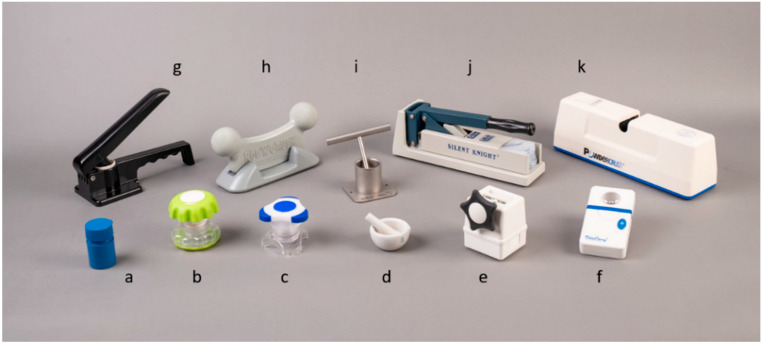
Tablet-crushing devices used in the study, listed in order of increasing cost of purchase: (**a**) Deluxe tablet crusher, (**b**) Ergo-Grip tablet crusher, (**c**) Tri-Grip tablet crusher, (**d**) mortar and pestle, (**e**) MiniTwist Quiet Crusher, (**f**) VitaCarry electronic pill grinder, (**g**) Ocelco plastic pill crusher, (**h**) Roc N Crush, (**i**) ball and socket tablet pulveriser, (**j**) Silent Knight pill crusher, and (**k**) Powdercrush electronic crusher. Further details of these crushers are available in Thong et al., 2018 [17].

A total of 11 tablet-crushing devices were involved in this study (Figure 1). These were selected from the 24 devices previously assessed for drug loss [17] as being representative of the variation in the device operation. Deluxe, Ergo-Grip, Tri-Grip, and MiniTwist were manual devices that were operated by hand-twisting to crush the tablet. The mortar and pestle and the ball and socket devices crushed the tablet with a repeated crushing action within a bowl. Roc N Crush was operated by rocking the top component to crush the tablet placed underneath. The Ocelco plastic crusher with cups and the Silent Knight crusher with bags were operated by lifting the lever up and down to exert a crushing pressure on the crushing pad. The VitaCarry electronic pill grinder and the Powdercrush electronic crusher with bags were operated by pressing a button. MiniTwist, Silent Knight, and Powdercrush required the tablet to be placed into a specific disposable plastic bag and inserted into the device whereas the Ocelco plastic crusher required a tablet to be placed between two disposable cups [17].

Each participant tested nine devices. Two devices, the MiniTwist and the VitaCarry electronic grinder (Figure 1e,f) malfunctioned during the first phase of data collection with the participants without limited hand functions. These were replaced with a Tri-Grip and ball and socket tablet pulveriser (Figure 1c,i) for the participants with limited hand functions as it was not possible to replace the malfunctioning devices (which are not available in Australia and had been purchased from overseas suppliers) with similar products.

Each participant crushed one paracetamol 500 mg tablet (Panamax, Sanofi Aventis Australia, Macquarie Park, NSW) in each of the nine crushing devices in a random order. Where instructions accompanied the product (e.g., on the packaging or a leaflet insert), these were provided to the participant with the product. After using each crusher, the participants completed the Rapid Assessment of Product Usability and Universal Design (RAPUUD) questionnaire [21]. The RAPUUD contained 12 questions on a 5-point Likert scale, ranging from ‘strongly disagree’ to ‘strongly agree’ (Supplementary Materials). Two questions were altered slightly in their wording to make them more appropriate for Australian consumers: the word ‘repeat’ replaced ‘do over’ in question 5, and the word ‘have’ replaced ‘get’ in question 6. A usability score was calculated for each tablet-crushing device from the twelve questions coded on a one-to-five scale, where five indicated a better usability and one indicated a poorer usability [21]. For positively worded questions (questions 1–3, 7, 9, and 10), ‘strongly agree’ was assigned a score of five and ‘strongly disagree’ was assigned a score of one. For negatively worded questions (questions 4–6, 8, 11, and 12), ‘strongly disagree’ was assigned a score for five whereas ‘strongly agree’ was assigned a score of one. The scores were summed, giving a maximum possible usability score of 60. If ‘not applicable’ was selected as the answer to a question, it was excluded from the calculation of the total scores for that tablet-crushing device and the score was normalised to a maximum of 60. All scores were then normalised to lie in the range of 0 to 100.

Two additional questions were asked regarding the likelihood of purchasing the tablet-crushing device, with answers on a 5-point scale (likely, somewhat likely, neither likely or unlikely, somewhat unlikely, and unlikely), and the maximum value that the participants would be willing to pay if they decided to purchase that tablet-crushing device. All questionnaires were piloted on two pharmacy students, two non-pharmacy students, one pharmacist and one older adult for understanding.

After all nine crushers had been tested, participants were asked to rank their top three tablet-crushing devices based on their ease of use. They were then shown the cost to obtain each device and asked to rank their top three devices again. These ranks were converted to scores by awarding 3 points to the first preference, 2 points to the second preference, and 1 point to the third preference.

### 2.4. Statistical Analysis

The statistical analysis was performed using GraphPad Prism 7. The dynamometer reading and AIMS2 scores were tested for a Gaussian distribution. As the data passed the D’Agostino and Pearson normality test (*p* = 0.142), the Pearson two-tailed correlation was performed to calculate the correlation coefficient (r) and the coefficient of determination (R^2^). The dynamometer readings were separated into eight groups according to different combinations of the three variables of gender, dominant/non-dominant hand, and with or without a self-reported limitation of hand functions. These data were normally distributed. An ANOVA with 12 pre-selected multiple comparisons with Sidak’s correction was performed to investigate whether there were differences in the dynamometer readings between males and females, between dominant and non-dominant hands, and between those with or without a self-reported limitation of hand functions.

The usability scores for each crushing device within the two groups of participants with and without limited hand functions using nine crushers were tested for normality. Thirteen of the eighteen groups of data passed the normality test. The standard deviations in the non-normally distributed groups were not significantly different according to the Brown–Forsythe test. Therefore, an ANOVA was used for the statistical comparisons as it is recognised to be a reasonably robust method in these situations. For each separate group of participants (i.e., those with limited hand functions and those without limited hand functions), a separate one-way ANOVA was also performed to compare the mean usability values of each crusher within the group of nine tablet-crushing devices tested. Post hoc multiple comparisons using the Tukey test were made between the mean usability score for each crusher and the mean of every other crusher. For each of the crushers tested by all participants, a one-way ANOVA was followed by pre-selected post hoc comparisons with Sidak corrections to compare the mean usability score given by those with limited hand functions versus those without limited hand functions. The rank scores awarded for the top three crushers were investigated with a non-parametric Kruskal–Wallis test followed by a Dunn’s multiple comparison of the pre-selected scores in order to compare the mean rank before and after being informed of the cost of each device.

## 3. Results

A total of 100 participants were recruited for this study. Of these, 60 participants self-reported being without limited hand functions whereas 40 reported having limited hand functions (Table 1). The most common conditions reported by those with limited hand functions were rheumatoid arthritis (30.0%), strokes (27.5%), osteoarthritis (12.5%), and physical injuries (12.5%). The age distribution for the participants without limited hand functions was skewed towards a younger age compared with the participants with limited hand functions, which was skewed towards mid and older ages (Table 1). Only 5% of the participants reported that they crushed tablets in order to swallow them (Table 1).

**Figure 2:**
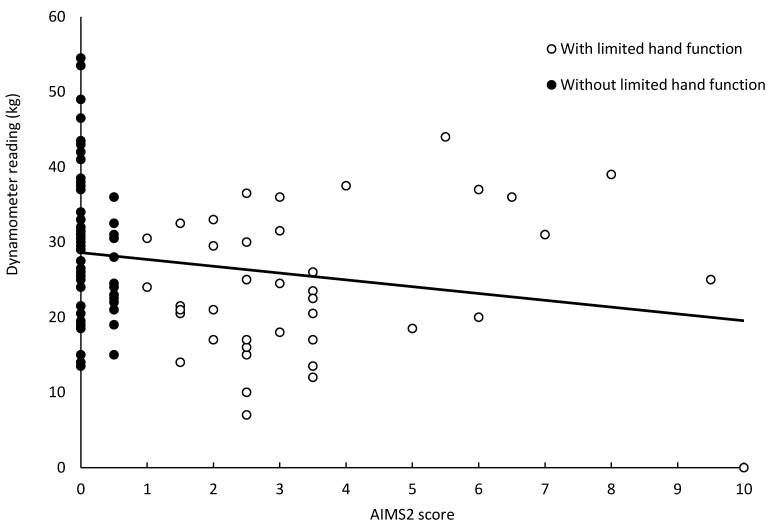
Coplot of the Arthritis Impact Measurement Scales version 2 (AIMS2) score for hand function against dynamometer reading for grip strength for the dominant hand. Participants self-reported having (open circles) or not having (filled circles) a condition or injury that affected hand functions. The regression line through the data for all 100 participants demonstrates r = −0.1998 and R^2^ = 0.0399.

The dynamometer measurements ranged from 14 to 55 kg for the dominant hand of the participants without limited hand functions, and 7 to 44 kg for participants with limited hand functions (Table 2). Although these ranges were wide and overlapping, the average dynamometer reading of participants with limited hand functions was significantly lower than that of participants without limited hand functions (*p* < 0.05). The readings were lower for females than males (*p* < 0.05), but there was no difference between dominant and non-dominant hands. The AIMS2 scores more clearly differentiated the participants with limited hand functions (AIMS2 scores from 1 to 10) from those who reported no limitation of hand functions (AIMS2 scores of 0 and 0.5). However, there was no correlation between the dynamometer reading and AIMS2 score (R^2^ = 0.0399) (Figure 2).

**Figure 3:**
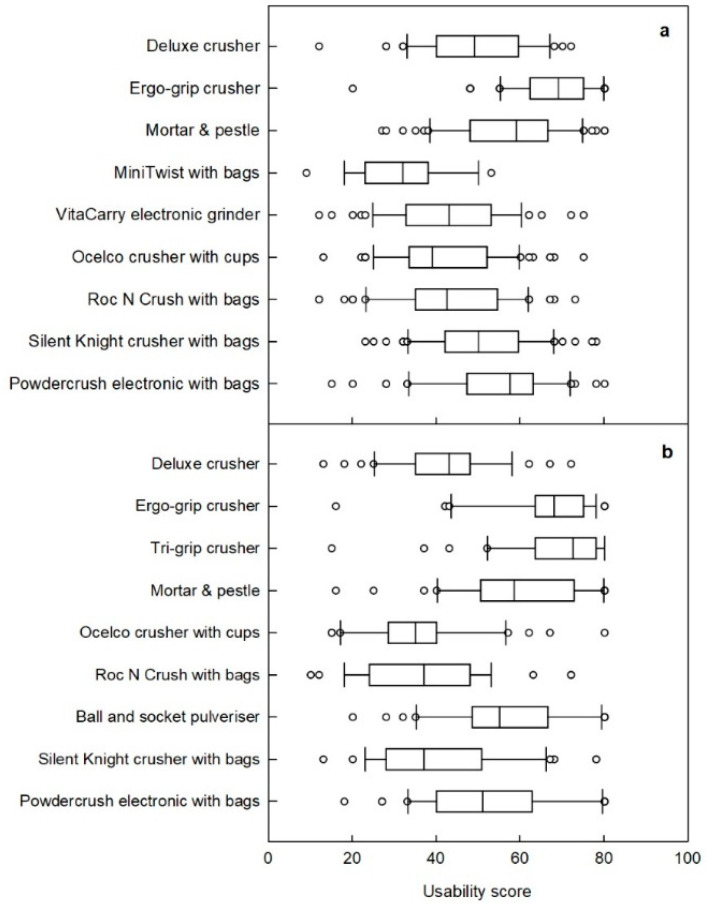
Box and whiskers graph for tablet crushers tested by participants (**a**) without limited hand functions (*n* = 60) and (**b**) with limited hand functions (*n* = 40). Scores were obtained from the responses to 12 questions from the RAPUUD questionnaire, normalised to lie in the range of 0 to 100 with a higher score indicating better usability [21]. The box extends from the 25th to the 75th percentiles, the vertical line in the box is the median, the whiskers indicate the 10th and 90th percentiles, and open circles are outliers. The MiniTwist with bags and VitaCarry electronic grinder malfunctioned during testing by participants without limited hand functions and were replaced with the Tri-Grip and ball and socket devices for participants with limited hand functions.

When the crusher usability was tested by the participants without limited hand functions (Phase 1), the Ergo-Grip scored the highest, with a mean score of 67.7 out of 100, which was significantly better than all the other tablet-crushing devices (Figure 3a). At the other end of the scale, the MiniTwist with bags was only used by 19 participants before it malfunctioned. Even whilst functional, it obtained the lowest average usability score, with a mean score of 31.9 (range: 9–53) due to difficulty twisting the handle and the need to repeat the function multiple times to crush the tablet (Figure 3a). The VitaCarry electronic pill grinder stopped working after 58 participants and although it received a stronger usability score (42.8) than the MiniTwist with bags, it was rated as having lower usability than 4 of the other crushers (Table 3). The VitaCarry electronic grinder was viewed as being difficult to clean after use and not appropriate to share between users.

Among the participants with limited hand functions, the Ergo-Grip and the similarly designed Tri-Grip crushers obtained the highest average usability scores, with mean scores of 66.2 and 68.0, respectively (Figure 3b; Table 3). The four tablet crushers that obtained the lowest mean scores in this group were the Deluxe tablet crusher, Roc N Crush with bags, Ocelco crusher with cups, and Silent Knight crusher with bags (Table 3). Comments provided by a few participants with limited hand functions reflected that they faced difficulty in opening and getting a tablet into the individual disposable bags. This affected their usability scores, even though the crusher itself may have been easy to operate.

Of the seven devices that were tested by all participants, only the mean score for the Silent Knight crusher with bags was significantly different, having a lower usability for participants with limited hand functions than those without limited hand functions (Table 3). The usability scores of the other six devices were comparable between the groups of participants.

**Figure 4:**
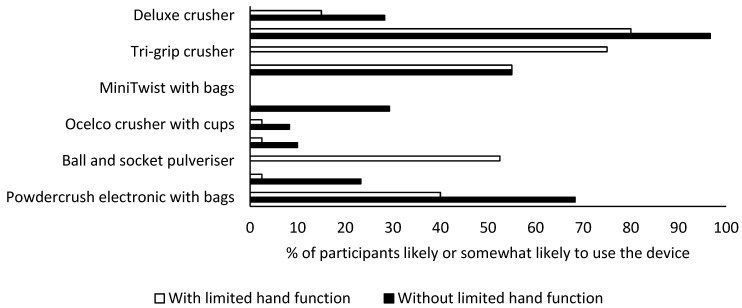
Proportion of participants with or without limited hand functions who considered they would be likely or somewhat likely to select each crusher after completing the RAPUUD for each device. Devices were tested by 60 participants without limited hand functions and 40 participants with limited hand functions. The MiniTwist with bags and VitaCarry electronic grinder malfunctioned during testing by participants without limited hand functions and were replaced with the Tri-Grip and ball and socket for participants with limited hand functions.

More than half of the respondents without limited hand functions reported that they would be likely or somewhat likely to use the Ergo-Grip (58/60; 97%), the Silent Knight electronic crusher with bags (41/60; 68%), and a mortar and pestle (33/60; 55%) (Figure 4). The participants with limited hand functions also reported that they would be likely or somewhat likely to use the Ergo-Grip (32/40; 80%) or the similarly designed Tri-Grip crusher (30/40; 75%). They also indicated positively towards the mortar and pestle (22/40; 55%) and the ball and socket (21/40; 53%) (Figure 4).

**Figure 5:**
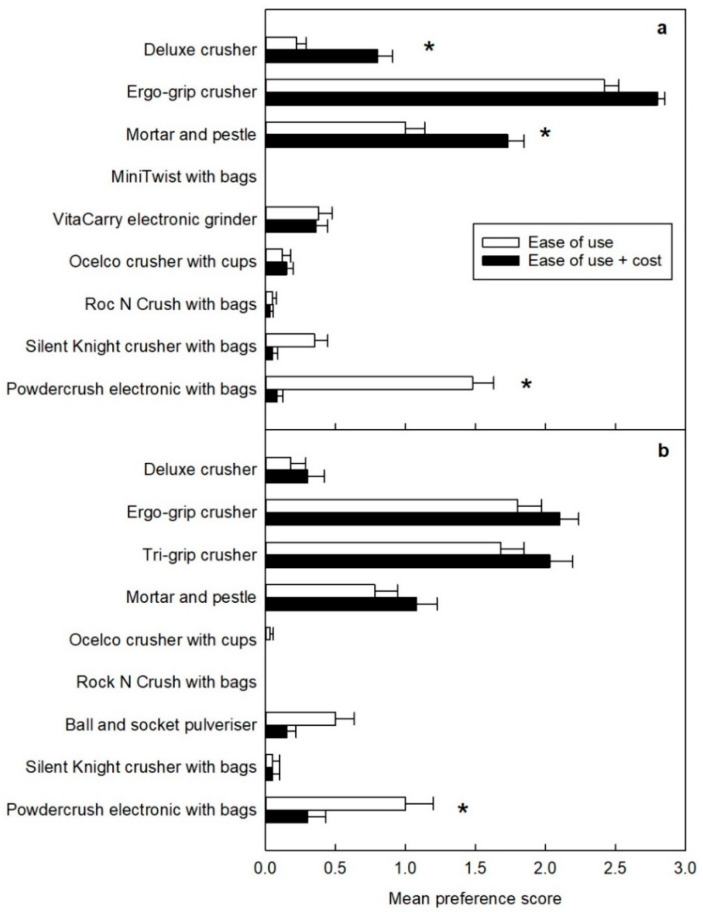
Mean score of each tablet-crushing device by participants (**a**) without limited hand functions and (**b**) with limited hand functions. For each participant, the tablet-crushing device rated as first choice was awarded 3 points, 2 points for second choice, and 1 point for third choice. Ratings were based on ease of use before (white bars) or after (black bars) the participant was provided with the approximate cost of each device. A significant difference (*p* < 0.05) in mean score without and with a consideration of cost is indicated by *. Devices were tested by 60 participants without limited hand functions and 40 participants with limited hand functions. The MiniTwist with bags and VitaCarry electronic grinder malfunctioned during testing by participants without limited hand functions and were replaced with the Tri-Grip and ball and socket for participants with limited hand functions.

When participants chose their favourite three crushers based on ease of use, the Ergo-Grip received the highest average score across both groups, irrespective of whether the cost was considered or not (Figure 5). The Tri-Grip crusher was tested by the group of participants with limited hand functions and it also scored highly (Figure 5b). The Powdercrush electronic crusher with bags came next in the rankings by both groups of participants when based purely on ease of use, but the score significantly dropped (Figure 5) when participants became aware of the cost of the crusher (> AUD 200). After introducing the cost of the devices, the participants without limited hand functions replaced the Powdercrush electronic crusher with bags with the Deluxe crusher and the mortar and pestle (AUD 5–20), as evidenced by the significant increase in the score awarded (Figure 5a). The participants with limited hand functions were less consistent in their replacement; all of the cheaper crushers received more points, but no single item exhibited a significant increase in score (Figure 5b).

**Figure 6:**
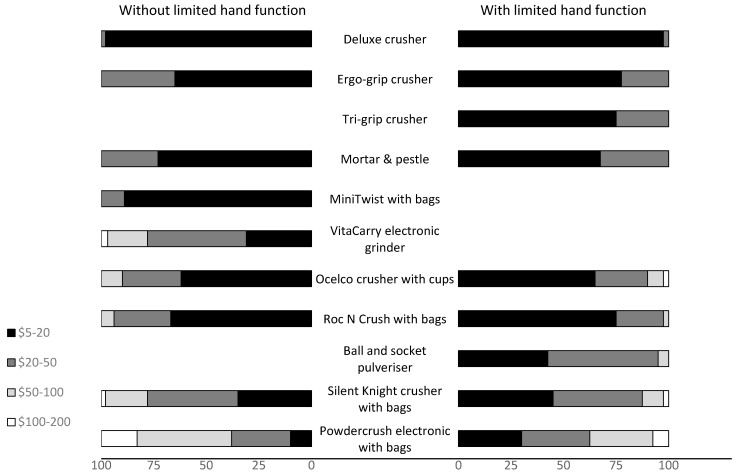
The maximum amount participants without limited hand functions and with limited hand functions were willing to pay for each tablet-crushing device. The percentages of participants choosing the price category for each device are reported. No participant was willing to pay more than AUD 200 for a tablet-crushing device.

Fewer than 20% of the participants from both groups were willing to pay more than AUD 100 for any of the tablet-crushing devices (Figure 6). The significant drop in selection within the top three (Figure 5) that occurred for the Powdercrush electronic crusher with bags after the actual cost of the device was revealed (AUD 500–600) was not surprising, given that none of the participants indicated that they would be willing to pay more than AUD 200 for it (Figure 6).

## 4. Discussion

The findings of the study indicated that certain pill crushers were more preferred in terms of their usability by people with and without limited hand functions. Overall, the economical twist-action crushers that were designed to improve the hand grip (such as the Ergo-Grip and Tri-Grip crushers) were found to have the best usability by participants with and without limited hand functions. Patients with severe forms of rheumatoid arthritis with finger joint stiffness find it difficult to grip and perform twisting movements such as opening jars [23,24]. However, our participants with rheumatoid arthritis still indicated that they would be likely to use the Ergo-Grip or Tri-Grip twist as their preferred device. The ergonomic design of the handles was important, given that the simpler basic twist device (the Deluxe tablet crusher) was given a significantly lower usability score and most of the participants with limited hand functions due to arthritis indicated that that they would be unlikely to select it.

Although the preferences of participants leaned towards certain products, the range of usability scores was large for almost all devices, irrespective of whether the participant group had a limited hand function or not. This indicated that no single crusher design suits everyone, even when the hand function is not a limitation. Different tablet-crushing devices on the market that differ in characteristics offer a range of options for consumers to choose one that suits them best.

The small number of participants and the single-centre nature of the study constrained the statistical power and limited the ability to extrapolate the findings more widely [21]. Additionally, a single, uncoated, immediate-release tablet was tested in the present study; the muscular demand to use a few devices increases when more tablets are crushed [18]. Moreover, the effort needed to crush different types of tablets can vary, depending on the physical properties (such as porosity, hardness, or coating type) [12,25] and, therefore, may affect the usability scores. The time taken to undertake each task as well as the fatigue associated with using multiple products should also be considered in future investigations.

The overall age distribution of the sample was relatively younger than the market target for tablet-crushing devices, although it can be assumed that usability issues can affect both young and elderly users. Healthy participants without limited hand functions were younger compared with the participants with limited hand functions; age-matching was not attempted in this study. Indeed, diseases such as arthritis and strokes that affect hand functions tend to affect older people. Community-dwelling older people who often manage their medications themselves have reported a range of problems in handling their medications such as opening pill bottles and crushing or breaking tablets [26]. Older people with highly complex medication regimens, which may require the medications to be modified, are more likely to have impaired dexterity [27]. Older people are also more likely to experience swallowing difficulties due to age-related physiological changes or as a sequela of comorbidities. As a result, many older people tend to crush their pills to facilitate swallowing [28]. However, even though the groups were not age-matched, the usability scores were similar between the groups for most of the tablet-crushing devices, with only the manual crusher with bags (Silent Knight) being significantly less usable for those with limited hand functions.

The usability of tablet-crushing devices was assessed in the present study from the point of view of personal use by people with and without limited hand functions. The devices preferred in this study are relevant for individuals who modify their medications at home, but may be impractical for staff in hospitals or aged-care facilities, especially when a large number of medications need to be modified and delivered. The results would be expected to differ if tested by nurses and other healthcare workers involved in hospital or aged-care medication delivery. Tablet-crushing devices are commonly shared among residents of aged-care facilities and not cleaned between each resident [5,7,29]. This results in the left-over drug powder being unintentionally given to other residents, which can cause significant drug interactions [5,7] and adverse effects such as hypersensitivity reactions [6,30]. Tablet-crushing devices with self-contained disposable bags or cups offer advantages in healthcare settings as they reduce the risk of cross-contamination and the need to routinely clean the tablet-crushing device, especially when the device is shared among different individuals [7]. Indeed, manual and electronic crushers with bags are the two most commonly used types of crushing devices in aged-care facilities [7]. A difficulty crushing tablets was expressed as one of the problems in managing medications by 12.5% of caregivers in care homes [31]. The repeated and long-term use of devices that are not comfortable or ergonomic can potentially increase the risk of healthcare workers developing work-related musculoskeletal problems [18]. Future studies should, therefore, explore the opinions of healthcare workers in different healthcare settings about the usability and preferences of tablet-crushing devices.

## 5. Conclusions

The findings from this study provide an insight into the thoughts and opinions of people on the usability of a range of designs for tablet-crushing devices. The economical twist-action crushers without separate bags or cups such as the Ergo-Grip and Tri-Grip were generally found to have a greater usability and were preferred by community-dwelling participants with and without limited hand functions. Usability may not be the only consideration during the pill crusher selection process by consumers; in this study, the cost of the devices also influenced the preference of tablet crushers. The efficiency and effectiveness of crushing as well as powder losses during crushing and transfer must also be considered, along with the importance of avoiding cross-contamination between users. The findings of this study can be used by consumers to better select their crushing device and by manufacturers to improve the crusher design. Usability testing of administration devices should be performed with all relevant subsets of the older adult population, paying attention to the specific human factors of each subset, including their physical, sensory, emotional, and intellectual capabilities.

## Figures and Tables

**Table 1:** Demographics of participants surveyed (*n* = 100), with those who self-reported having an injury or condition that caused a limited hand function separated from those without a limited hand function. Data show the number of participants with % in brackets.

Demographic	Without Limited Hand Functions (*n* = 60)	With Limited Hand Functions (*n* = 40)
Gender		
Male	17	18
Female	43	22
Age group (years)		
18–29	32 (53%)	4 (10%)
30–45	16 (27%)	0 (0%)
46–60	11 (18%)	17 (42%)
61–75	1 (2%)	14 (35%)
76–90	0 (0%)	5 (13%)
Taking regular or occasional medications		
Yes	29 (48%)	34 (85%)
No	31 (52%)	6 (15%)
Crush pills before swallowing them?		
Yes	2 (3%)	3 (7%)
No	58 (97%)	37 (93%)

**Table 2:** Measurements for hand functions of study participants. The physical domain questions from the revised version of the Arthritis Impact Measurement Scales version 2 (AIMS2) were used to calculate a hand function score [22]; a score of 0 represented good hand and finger functions whereas a score of 10 indicated poor hand and finger functions. Squeezing a hydrolytic hand dynamometer was used to assess grip strength for each hand, with the participants self-identifying their dominant and non-dominant hand. The average of two measurements for each hand for each participant was used in the calculations.

Question	Without Limited Hand Function (*n* = 60)		With Limited Hand Function (*n* = 40)	
Hand dominance	*n*	%	*n*	%
Right	57	95%	37	93%
Left	3	5%	3	7%
Dominant hand grip strength (kg)	Range	Mean ± SE	Range	Mean ± SE
Male	24.5–54.5	40.1 ± 8.0	7.0–44.0	28.9 ± 10.3
Female	13.5–38.0	25.4 ± 6.2	12.0–32.5	19.8 ± 7.0
Non-dominant hand grip strength (kg)	Range	Mean ± SE	Range	Mean ± SE
Male	27.5–50.5	38.2 ± 6.3	4.0–36.5	22.8 ± 10.0
Female	12.5–37	23.8 ± 5.7	4.0–24.5	15.3 ± 6.6
AIMS2 score	Range	Median	Range	Median
Male	0–0.5	0	1–9.5	3
Female	0–0.5	0	1–10	3

**Table 3:** Usability scores provided by participants with or without limited hand functions for different tablet crushers. Scores were obtained from the responses to 12 questions from the RAPUUD questionnaire, normalised to lie in the range of 0 to 100, with a higher score indicating better usability [21]. Devices were tested by 60 participants without limited hand functions and 40 participants with limited hand functions. The MiniTwist with bags and VitaCarry electronic grinder malfunctioned during testing by participants without limited hand functions and were replaced with the Tri-Grip and ball and socket for participants with limited hand functions. Within each group of participants (each column), the devices that share the same letter were not significantly different. A significant difference between groups is indicated by * after the crusher name.

Crusher	Without Limited Hand Functions (*n* = 60)		With Limited Hand Functions (*n* = 40)	
	Mean ± SE		Mean ± SE	
Deluxe tablet crusher	49.2 ± 1.65	a,e	42.4 ± 2.08	a
Ergo-Grip tablet crusher	67.7 ± 1.37	b	66.2 ± 2.07	b
Tri-Grip pill crusher			68.0 ± 2.13	b
Mortar and pestle	57.3 ± 1.66	d	58.6 ± 2.43	b,d
MiniTwist with bags	31.9 ± 2.55	c		
VitaCarry electronic grinder	42.8 ± 1.81	e		
Ocelco crusher with cups	41.5 ± 1.66	c,e	36.0 ± 2.23	a
Roc N Crush with bags	43.3 ± 1.81	e	37.3 ± 2.35	a
Ball and socket pulveriser			55.8 ± 2.26	c,d
Silent Knight crusher with bags *	50.7 ± 1.62	a,d	39.8 ± 2.46	a
Powdercrush electronic crusher with bags	54.9 ± 1.76	a,d	52.5 ± 2.48	c,d

## Data Availability

The data presented in this study are available on request from the corresponding author. The data are not publicly available due to privacy considerations.

## Supplementary Materials

The following supporting information can be downloaded at: https://www.mdpi.com/article/10.3390/pharmaceutics15020517/s1. File S1: Usability of pill crushers.
