# Supplementary material for: Perceived Usability of Tablet Crushers: Comparison of Devices by People with and without Limited Hand Functions
Source: Pharmaceutics. 2023 Feb 3;15(2):517. doi: 10.3390/pharmaceutics15020517 (PMC9961238; doi:10.3390/pharmaceutics15020517)
Supplement: Supplementary file 1 [file pharmaceutics-15-00517-s001.zip › pharmaceutics-2103680-supplementary.pdf]

Participant number:

THE UNIVERSITY OF QUEENSLAND
AUSTRALIA

Usability of pill crushers

PART A. Demographic Information

1. What is your age:__________________

2. What is your gender? (please circle one answer)

Female	Male	Other

3. What is your highest level of education? (please circle one answer)

No formal qualifications	Year 10 or equivalent	Year 12 or equivalent	Trade or apprenticeship
Certificate of Diploma	University degree	Higher University degree	Research Higher Degree

4. What is your approximate current personal income? Please include the total of all wages, salaries, benefits, pensions, allowances and any other income you usually receive, before deductions for tax, superannuation, insurance etc. (Please select one range)

**Weekly personal income (annual personal income)**	
Negative income	
Nil income	
$1-$299 ($1-15,599)	
$300-$599 ($15,600-$31,199)	
$600-$999 ($31,200-$51,999)	
$1,000-$1,499 ($52,000-$77,999)	
$1,500-$1,999 ($78,000-$103,999)	
$2,000 or more ($104,000 or more)	

Please turn over for more questions

5. Do you have any medical conditions that impact the flexibility, strength or movement of your hands? (please circle one answer)

Yes	No

If yes, please name the medical condition____

6. Do you take any regular or occasional medications (e.g. prescription, over the counter, vitamins or herbal medicines)? (please circle one answer)

Yes	No

7. Do you crush pills before you swallow them? (please circle one answer)

Yes	No

If yes, how do you crush them? ____

8. During the past month (please select one answer to each question)

	All days	Most days	Some days	Few days	No days
Could you easily write with a pen or pencil?					
Could you easily button a shirt or blouse?					
Could you easily turn a key in a lock?					
Could you easily tie a knot or a bow?					
Could you easily open a new jar of food?					

9. Dynamometer reading _______________________kg

THE UNIVERSITY OF QUEENSLAND
AUSTRALIA

Crusher name:	Participant number:

PART B. Pill Crusher Specific Questions

1. For the crusher you just used please rate the following questions:

This is a standard questionnaire that is designed for assessing product usability, please select one answer to each question.

	Strongly Disagree	Somewhat Disagree	Neutral	Somewhat Agree	Strongly Agree	N/A
This product is easy to set-up or prepare to use						
This product is easy to use						
This product is easy to clean-up and place into storage						
For me, this product poses a safety risk						
I need assistance to use this product						
When using this product I make mistakes or errors that require me to repeat some steps						
I have the information I need to use the product efficiently						
Using this product takes more time than it should						
Using this product requires little physical effort						
Using this product requires minimal mental effort						
Using this product draws unwanted attention to me						
I feel embarrassed when using this product						

Please turn over for more questions

2. a) If you needed to crush your pills, how likely would you be to use this pill crusher? (please circle one answer)

Unlikely	Somewhat Unlikely	Neither Likely or Unlikely	Somewhat Likely	Likely

b) What is the maximum that you would <u>be willing to pay</u> if you decided to purchase one of these? (please circle one answer)

$5-20	$20-50	$50-100	$100-200	$200-300	$300-400	$400-500	$500-600

Participant number:

THE UNIVERSITY OF QUEENSLAND
AUSTRALIA

PART C. Final Overall Questions

1. Overall, select your top three pill crushers based on ease of use (number your top 3)

Note that images have been removed due to copyright; please refer to the manuscript.

**Basic twist** ☐	**Mortar and Pestle** ☐	**Crusher with cups** ☐
**Ergonomic twist** ☐	**Roc n Crush** ☐	**Crusher with bags** ☐
**Minitwist with bags** ☐	**Electronic grinder** ☐	**Electronic crusher with bags** ☐

2. Overall out of all of the pill crushers you have used, which would you choose to purchase if you needed one and why?

Which one would you choose?____

Why?____

Please turn over for more questions

3. The approximate price range that you could expect to pay for one of these crushers is given in Australian dollars inclusive of shipping.

   With all things considered, if you decided to purchase a pill crusher which would be your top 3 choices? (number your top 3)

Note that images have been removed due to copyright; please refer to the manuscript.

**Basic twist ($5-15)**	**Mortar and Pestle ($10-20)**	**Crusher with cups ($80-100)**
**Ergonomic twist ($10-20)**	**Roc n Crush ($100-150)**	**Crusher with bags ($200-300)**
**Minitwist with bags ($40-50)**	**Electronic grinder ($60-80)**	**Electronic crusher with bags ($500-600)**
